# Upper Critical Field and Tunneling Spectroscopy of Underdoped Na(Fe,Co)As Single Crystals

**DOI:** 10.3390/ma16196421

**Published:** 2023-09-27

**Authors:** Leonid Morgun, Svetoslav Kuzmichev, Igor Morozov, Alena Degtyarenko, Andrey Sadakov, Andrey Shilov, Ilya Zhuvagin, Yevgeny Rakhmanov, Tatiana Kuzmicheva

**Affiliations:** 1 Lebedev Physical Institute, Russian Academy of Sciences, 119991 Moscow, Russia; morgunla@lebedev.ru (L.M.); kuzmichev@mig.phys.msu.ru (S.K.);; 2 Faculty of Physics, Lomonosov Moscow State University, 119991 Moscow, Russia; 3 Department of Chemistry, Lomonosov Moscow State University, 119991 Moscow, Russia

**Keywords:** unconventional superconductivity, alkali-metal pnictides, single-crystal growth, magnetotransport, upper critical field, tunneling spectroscopy, Andreev spectroscopy

## Abstract

A comprehensive study of superconducting properties of underdoped NaFe${}_{0.979}$Co${}_{0.021}$As single crystals by a combination of upper critical field measurements and incoherent multiple Andreev reflection effect (IMARE) spectroscopy is presented. The $H_{c2}\left(T\right)$ temperature dependences are measured at magnetic fields up to 16 T with in-plane and out-of-plane field directions and considered within single-band and two-band models in order to estimate the $H_{c2}\left(0\right)$ value. In IMARE spectroscopy probes, the magnitude, characteristic ratio, and temperature dependence of the superconducting order parameters ($\Delta_{L,S}\left(T\right)$) are determined locally and directly. A possible *k*-space anisotropy of the large superconducting gap is demonstrated. We show that usage of a quadruple of $\lambda_{ij}^{0}$ coupling constants obtained in the IMARE experiment can significantly reduce the number of free parameters required to fit the $H_{c2}\left(T\right)$ dependence within a two-band approach (from six to two).

## 1. Introduction

Layered pnictides Na(Fe,Co)As relate to the so-called 111 family of the iron-based superconductors [1,2]. The 111 family of alkali-metal-based superconducting (SC) pnictides, with LiFeAs and NaFeAs as representative members, has attracted the attention of theoreticians and experimenters due to its unique set of properties that are not typical of other families of iron-based SC (for a review, see [3]). For example, NaFeAs shows superconductivity even in a stoichiometric state with rather low $T_{c}\approx 10$ K [4,5]. Above this $T_{c}$, antiferromagnetic (AFM) and nematic phases develop at $T_{m}\approx 43$ K and $T_{s}\approx 55$ K, respectively. Under a partial substitution of Fe by transition metal (Tm) [3], the critical temperature of NaFe${}_{1-x}$Tm${}_{x}$As reaches a maximum of $T_{c}\approx 22$ K simultaneously with AFM and nematicity suppression. A number of probes [6,7,8,9] show a natural phase separation in underdoped NaFe${}_{1-x}$Tm${}_{x}$As, with clusters of tetragonal SC phase and orthorhombic AFM phase coexisting in the bulk single crystal. With electron doping, the volume fraction of AFM clusters gradually decreases, whereas the optimally doped NaFe${}_{1-x}$Tm${}_{x}$As crystal is fully occupied by the SC phase [6,7,8]. The Fermi surface of NaFe${}_{1-x}$Tm${}_{x}$As consists of hole barrels around the Γ point of the first Brillouin zone and electron barrels near the M point [10,11], where several SC condensates develop below $T_{c}$.

Owing to the difficulties in studying NaFeAs, there has been a lack of available experimental data to date. Due to the rapid (in a few minutes) degradation of NaFeAs in the presence of even trace amounts of H${}_{2}$O and O${}_{2}$, sample preparation and experiment should be conducted in a protective atmosphere.

Two-gap superconductivity in Na(Fe,Co)As was confirmed using surface probes (angle-resolved photoemission spectroscopy (ARPES) [11,12] and scanning tunneling spectroscopy [13,14]) and bulk techniques (specific heat measurements [4,5]). The magnitudes of the large and small SC gaps within the range of the characteristic ratios ($2\Delta \left(0\right)/k_{B}T_{c}\approx$ 5–9 and 2.5–4.5, respectively) were reported (see Figure 6 in [3]). The significant spread of the characteristic ratios of the SC gaps introduced in the Bardeen–Cooper–Schrieffer (BCS) theory and available in the literature to date obviously results from an absence of direct probes of the gap structure of Na(Fe,Co)As.

Two fundamental mechanisms of superconductivity have been suggested: spin-fluctuation Cooper pairing (so-called $s^{\pm }$ model) [15] and orbital-fluctuation pairing ($s^{++}$ model) [16]. Theoretically, the SC gap structure of NaFeAs could be described by both the $s^{++}$ [17] approach and the $s^{\pm }$ model [18,19]. $s^{\pm }$- and $s^{++}$-based calculations agree that the dependence of Cooper pair coupling energy ($2\Delta_{L}$) on the momentum direction (the SC gap anisotropy) in the $k_{x}k_{y}$ plane takes place in NaFeAs [17,19], as well as in iron-based superconductors with similar Fermi surface topology [20] and in sister LiFeAs compounds [21,22,23,24]. It is interesting to note that a valuable *k*-space anisotropy of the large SC order parameter was resolved in ARPES probes of underdoped Na(Fe,Co)As [12] that was unobserved by the same research group in overdoped crystals with similar $T_{c}\approx 18$ K. This fact could point to an influence of the AFM phase on SC properties.

Until now, the upper critical field temperature dependence of Na(Fe,Co)As has been studied in three works only: for compositions with a wide Co doping range [25,26] and for optimal and overdoped crystals [27]. While in [25,27], magnetotransport measurements were performed within the range of *H* = 0–9 T, in [26], the $H_{c2}\left(T\right)$ behavior was probed up to extremely high magnetic fields of about 45 T in parallel and perpendicular directions for Na(Fe,Co)As with various doping degrees. Unfortunately, the abundance of free parameters used in [26] to fit the experimental $H_{c2}\left(T\right)$ dependence with a two-band model (a quadruple of $\lambda_{ij}^{0}$ coupling constants with band diffusivities of $D_{1}$ and $D_{2}$), as well as the absence of a comprehensive study, could make the values of these free parameters ambiguous.

Here, a novel approach that provides a drastic decrease in the number of free parameters (from six to two) used in order to fit the experimental $H_{c2}\left(T\right)$ dependence for a multiple-gap superconductor is presented. The grown underdoped NaFe${}_{0.979}$Co${}_{0.021}$As single crystals are studied by a combination of upper critical field and incoherent multiple Andreev reflection effect (IMARE) spectroscopy probes. The $H_{c2}\left(T\right)$ dependences are measured at H≤16 T in two field directions: H‖ab and H‖c. Using IMARE, for the first time, we locally and directly determine the magnitudes and temperature dependences of the SC-order parameters and roughly estimate a quadruple of full coupling constants ($\lambda_{ij}^{0}$). The obtained $H_{c2}\left(T\right)$ temperature dependences are described using a two-band approach with estimated $\lambda_{ij}^{0}$ values.

## 2. Materials and Methods

**Figure 1:**
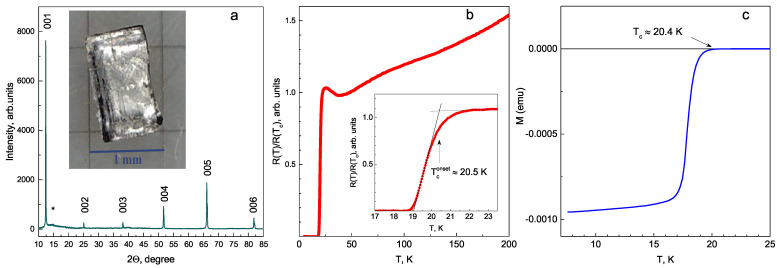
(**a**) XRD pattern of NaFe${}_{0.079}$Co${}_{0.021}$As single crystal. An image of a plate-like sample cut from a bulk crystal is shown in the inset. Minor feature possibly attributed to the parafilm substrate is marked by ${}^{*}$. (**b**) Temperature dependence of resistance. The inset details the SC transition below $T_{c}^{\mathrm{onset}}\approx 20.5$ K. (**c**) Magnetization SC transition with $T_{c}^{\mathrm{onset}}\approx 20.4$ K.

Single crystals of NaFe${}_{0.979}$Co${}_{0.021}$As of nominal composition were grown by crystallization from melt. All manipulations, such as obtaining the NaAs precursor and initial mixture preparation, as well as storage and preparation of samples for subsequent measurements, were performed in an argon-filled glove box with residual concentrations of water and oxygen less than 0.1 ppm. Then, preliminarily synthesized 0.058 g CoAs and 1.127 g Fe were added to 2.018 g of NaAs. The prepared reaction mixtures were placed in an alumina crucible, which was sealed in a niobium container in order to prevent a loss of alkali metal; then, the niobium container was sealed in an evacuated quartz ampoule. The ampoule was heated up to 1050 °C at a rate of 100 °C/h and held at this temperature for 24 h. Then, the ampoule was cooled down to 400 °C at a rate of 3 °C/h, annealed at this temperature for 24 h, and cooled down to room temperature in a turned-off oven. The grown single crystals were extracted mechanically from the ingot using a Levenhuk DTX 700 microscope (Tampa, FL, USA), then cut to a rectangular plate shape with dimensions of 1.5×2.5 mm${}^{2}$. The presence of a single SC phase was confirmed by X-ray diffraction (XRD), as well as resistive and magnetic measurements (Figure 1).

XRD studies were performed on a Rigaku MiniFlex 600 (Osaka, Japan) with CuKα radiation in the range of degrees of 5° to 85${}^{{}^{\circ}}\left(\Theta –2\Theta \right)$ with a scanning rate of 1° per minute and a step size of 0.02. The sample was placed in an air-sensitive holder in an argon glove box to prevent interaction with air. Figure 1a shows the XRD pattern for grown the NaFe${}_{0.079}$Co${}_{0.021}$As single crystal. All the observed peaks (00l) are sharp and pronounced, demonstrating the homogeneity of the grown crystal and are related to the P4/nmm tetragonal phase. An insignificant halo hump ${(}^{*})$ at an angle of 15° can be attributed to the parafilm substrate.

The bulk resistance of the NaFe${}_{0.079}$Co${}_{0.021}$As single crystal within wide temperature range obtained by a four-probe technique is shown in Figure 1b. The SC transition (detailed in the inset of Figure 1b) is observed below $T_{c}^{\mathrm{onset}}\approx 20.5$ K with a width of $\Delta T_{c}\approx 1.4$ K. Above $T_{c}$, the resistance of the NaFe${}_{0.979}$Co${}_{0.021}$As bulk single crystal falls with increased temperature and reaches a minimum at the temperature of the structural transition ($T_{s}\approx 36$ K), whereas at higher temperatures, R(T) shows a weak monotonic increase. For the studied crystals, the residual resistivity ratio is rather small: $R\left(200\;K\right)/R\left(T_{c}\right)\approx 1.5$. Magnetization M(T) measurements were performed using a SQUID-magnetometer MPMS-XL7 (Quantum Design, San Diego, CA, USA) in fields up to 5 Oe with H‖c. The resulting M(T) curve (Figure 1c) shows the SC transition at $T_{c}^{\mathrm{onset}}=20.4$ K. This critical temperature is similar to that determined by resistive measurements.

Magnetotransport probes were made using a cryogen-free measurement system (CFMS-16). The temperature dependence of the ab-plane resistivity ($\rho_{ab}$) was determined under a constant magnetic field of up to 16 T along both the ab plane and the *c* axis. The electric resistivity was measured using the standard four-probe AC lock-in technique with a current of 100 μA at a low frequency of 313.1 Hz. All Ohmic contacts were made using silver epoxy, ensuring a resistance of less than 10 Ω. To protect the sample from water vapor in the atmosphere, a thin layer of vacuum grease (Apiezon N, Clifton, NJ, USA) was applied to its surface.

**Figure A1:**
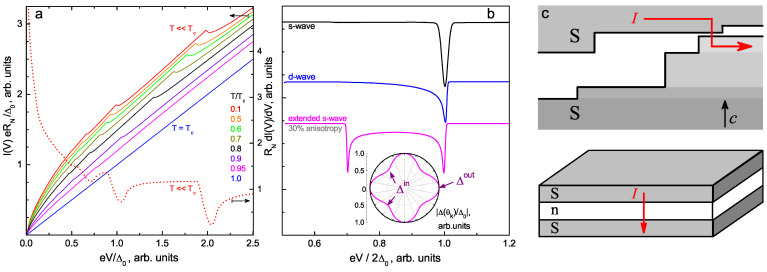
(**a**) Temperature evolution of the CVC of an SnS junction in the IMARE regime (left vertical axis) and the corresponding dynamic conductance spectrum at $T\ll T_{c}$ (right axis) calculated in the framework proposed in [28]. (**b**) Typical shape of an Andreev feature in the dI(V)/dV spectrum for the case of an isotropic SC gap with *s*-wave symmetry (black line), *d*-wave symmetry (blue line; the raw data are zoomed vertically by a factor of 10 for clarity), and extended *s*-wave symmetry with 30% anisotropy (magenta line). The data were numerically calculated using the approach proposed in [34]. For the latter case, the related SC gap angle distribution ($\Delta_{\theta }$) is shown in the inset. (**c**) Scheme of a planar break junction typically obtained in the layered single crystal.

In order to directly determine the SC gap structure, we used incoherent the multiple Andreev reflection effect (IMARE). The effect occurs in a symmetrical junction between two SC banks (S) separated by a relatively thin layer of normal metal (n), i.e., an SnS junction. The thickness of such a junction is d<l, where *l* is the inelastic scattering length and *d* is larger than the SC coherence length (so-called “long” junction). The current–voltage characteristic (CVC) and the dynamic conductance spectrum of the SnS junction in the IMARE regime predicted by [28] are shown in the Appendix A Figure A1a. Below $T_{c}$, the related CVC shows an excess current at all bias voltages as compared to the I(V) in the normal state [28,29,30,31]. The corresponding dynamic conductance (dI(V)/dV) spectrum shows an enhanced zero-bias conductance (ZBC) peak at eV→0 (so-called foot area) [28,30] and a series of dI(V)/dV dips named subharmonic gap structures (SGSs) [28,29,30,31,32,33]. The SGS positions ($eV_{n}\left(T\right)=2\Delta \left(T\right)/n$, where n=1,2,…) directly determine the magnitude of the SC-order parameter at any temperatures up to $T_{c}$ [28]. In the case of a multiple-gap superconductor, several SGS would appear in the dI(V)/dV spectrum.

Numerical calculations (see Figure A1b) based on the approach proposed in [34] have shown that in the case of an anisotropic but nodeless SC-order parameter with extended *s*-wave symmetry, each SGS subharmonic would represent a doublet; the positions of the two dI(V)/dV dips connected by an arch determine the maximum and minimum Cooper pair coupling energies in the *k*-space: $\Delta^{\mathrm{out}}$ and $\Delta^{\mathrm{in}}$, respectively. In particular, doublet-like Andreev features are reproducibly observed in the dI(V)/dV spectra of SnS junctions in NaFe${}_{0.079}$Co${}_{0.021}$As.

SnS junctions based on the Na(Fe,Co)As single crystals were formed using a mechanically controlled planar break junction (MCPBJ) technique [35]. The configuration of our experiment, as well as advantages and disadvantages of the technique, are reviewed in [36].

A layered NaFe${}_{1-x}$Co${}_{x}$As single crystal cut as a thin rectangular plate with typical dimensions of 2×1 mm${}^{2}$ was mounted onto a U-shaped springy sample holder parallel to the crystallographic ab plane using pads of liquid In-Ga alloy. The sample preparation and mounting were applied in a glovebox with a dry argon atmosphere in order to prevent degradation of the SC properties. Then, the holder was cooled down to T=4.2 K and precisely bent, cracking the crystal on two halves. In the sample, two cryogenic clefts with steps and terraces on the surface separated by a barrier were formed—a kind of superconductor–constriction–superconductor junction—whereas the measurement current always flows along the *c* direction (the scheme of the resulting junction is shown in Figure A1c). In NaFe${}_{1-x}$Co${}_{x}$As, the tunneling barrier typically obtained using the MCPBJ technique acts as thin normal metal with extremely high transparency of 80–95%, since the observed features of the resulting I(V) and dI(V)/dV curves resemble those predicted theoretically for a highly transparent IMARE regime [28,29].

Below, we summarize the advantages of IMARE spectroscopy of MCPBJ SnS contacts. The implemented technique provides direct local measurement of the magnitude of the SC gaps and their temperature dependences. Owing the clean formed cryogenic clefts, the determined SC gap magnitude is close to its bulk value. Using fine mechanical readjustment, it becomes possible to obtain dozens of SnS junctions with various $R_{N}$ values in one and the same sample during a single cooldown process. The latter facilitates collection of a large set of statistics, verifying the reproducibility of the data.

## 3. Results and Discussion

### 3.1. Upper Critical Field Probes

**Figure 2:**
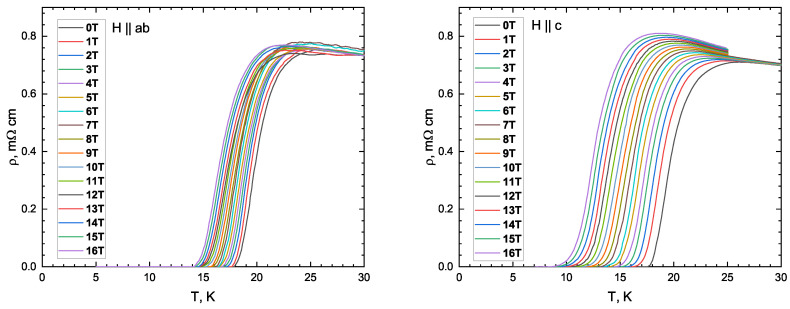
Evolution of temperature dependence of the in-plane resistivity ($\rho_{ab}$) for NaFe${}_{0.979}$Co${}_{0.021}$As with H‖ab and H‖c magnetic field directions. In both cases, the field direction is normal to the AC current direction.

**Figure 3:**
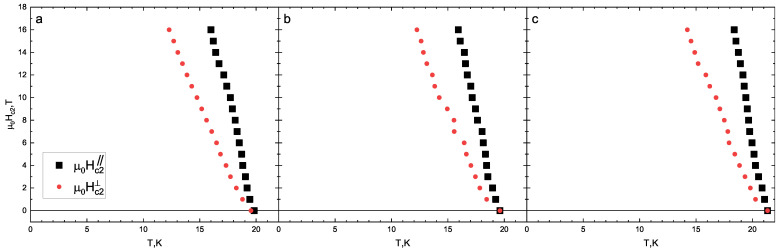
The maximum magnetic field (16 T) is definitely lower than the fundamental $H_{c2}\left(0\right)$. As a result, several criteria were employed to determine the $H_{c2}\left(T\right)$ temperature dependence in the vicinity of $T_{c}$, including the 50% of normal state resistivity criterion (**a**), the maximum dR/dT criterion (**b**), and the onset temperature ($T_{c}^{onset})$ criterion (**c**).

Figure 2 presents magnetotransport measurements in a static magnetic field for an underdoped NaFe${}_{0.979}$Co${}_{0.021}$As single crystal. The anomaly in the resistivity curve, namely an increase with a decrease in temperature, corresponds to the structural phase transition. The field-induced broadening of the SC transition is negligible for both directions of the magnetic field. In this study, three different criteria of SC critical temperature determination are used: (i) $T_{c}$ is taken as a temperature corresponding to a 50% resistance drop within the SC transition (hereafter, “50% criterion”); (ii) dR(T)/dT is taken as the maximum temperature (“max dR/dT” criterion); and (iii) the onset temperature of the SC transition is determined similarly to that shown in the inset of Figure 1b (“$T_{c}^{\mathrm{onset}}$” criterion). The obtained $H_{c2}\left(T\right)$ dependences with the temperature determined using the three above mentioned criteria are shown in Figure 3. Note that the obtained $H_{c2}\left(T\right)$ dependences are almost linear and show no “tails” in the close vicinity of $T_{c}$ (observed in Figure 7 in [25]).

According to ARPES measurements [11,37,38] and numerical calculations [39,40], the Na(Fe,Co)As compound is a quasi-2D superconductor with a Fermi surface consisting of collinear warped barrels. When a magnetic field is applied parallel to the SC planes, the electrons in the system form open orbits along the cylindrical Fermi surfaces. This results in a negligible orbital effect and a significant increase in the critical field above that expected for a more isotropic superconductor. On the other hand, when the magnetic field is applied perpendicular to the SC planes, closed electron orbits are formed within the Fermi surfaces, leading to the creation of vortices and reducing the orbital limit for the critical field. Therefore, in general, the parallel critical field ($H_{c2}^{\|}$) is greater than the perpendicular critical field ($H_{c2}^{⊥}$), as observed in our samples (see Figure 3).

In this paper, the studied samples are assumed to be in the dirty limit, where the mean free path ($l^{el}$) is relatively small and the coherence length (ξ) is of the same order of magnitude as $l^{el}$. This assumption is supported by the low residual resistivity ratio [41], the low superconducting volume fraction of the parent compound NaFeAs [7], and the tendency for pnictide superconductors to have a small mean free path ($l^{el}$) due to their low Fermi velocities [42] and high scattering rates. Unfortunately, the mean free path determination using Hall measurements could be ambiguous in the case of metal with multiple electron and hole bands. The average coherence lengths calculated for the studied samples are $\xi_{ab}\left(0\right)\approx 2.5$ nm and $\xi_{c}\left(0\right)\approx 2$ nm (see Table 1).

**Figure 4:**
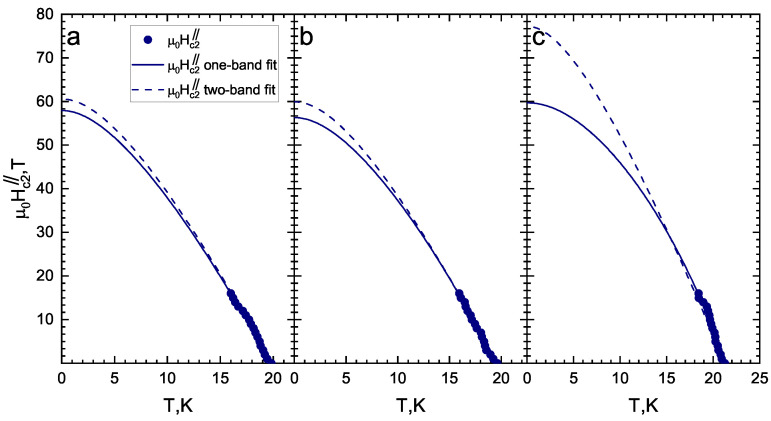
Temperature dependence of the upper critical fields ($H_{c2}^{\|}$) obtained in the framework of a single-band WHH model (solid lines) and two-band Gurevich model (dashed lines): (**a**) 50% criterion; (**b**) max dR/dT; (**c**) $T_{c}^{\mathrm{onset}}$.

**Figure 5:**
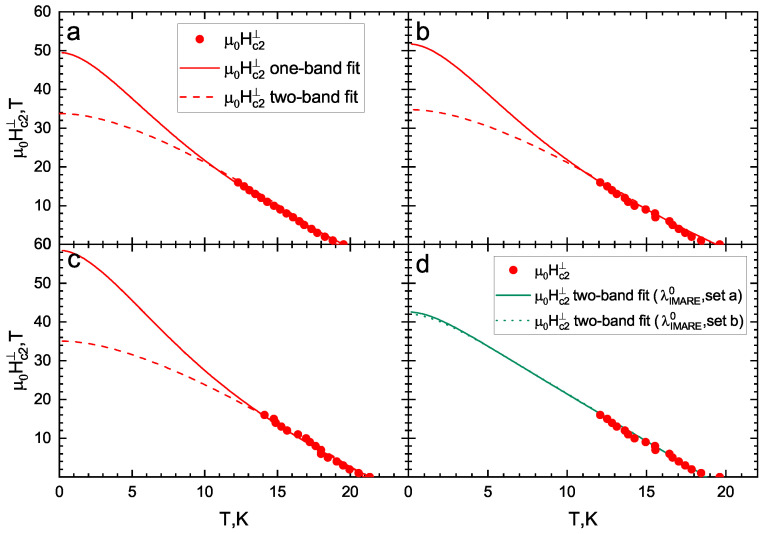
Temperature dependence of the upper critical fields ($H_{c2}^{⊥}$) obtained in the framework of a single-band WHH model (solid lines) and two-band Gurevich model (dash lines) with $\lambda_{11}^{0}=\lambda_{22}^{0}=1$ and $\lambda_{11}^{0}\lambda_{22}^{0}-\lambda_{12}^{0}\lambda_{21}^{0}=0.5$ taken from [26] under various critical temperature criteria: (**a**) 50% criterion; (**b**) max dR/dT; (**c**) onset of SC transition. (**d**) Temperature dependence of the upper critical field ($H_{c2}^{⊥}$) determined by max dR/dT and two-band Gurevich models with $\lambda_{11}^{0}=0.435$, $\lambda_{12}^{0}=0.236$, $\lambda_{21}^{0}=0.133$, and $\lambda_{22}^{0}=0.326$ (solid line) and $\lambda_{11}^{0}=0.412$, $\lambda_{12}^{0}=0.219$, $\lambda_{21}^{0}=0.108$, and $\lambda_{22}^{0}=0.304$ (doted line) (these two $\lambda_{ij}^{0}$ sets are derived from the renormalized $\lambda_{ij}$ estimated in the IMARE experiment: $D_{1}/D_{2}\approx 3.52$ and 3.75, respectively).

Using single-band Werthamer–Helfand–Hohenberg [43] and two-band Gurevich [44] models, the temperature dependencies of $H_{c2}$ were obtained as shown in Figure 4 and Figure 5. Generally, the two-band model in the dirty limit [44] has six free parameters: four “full” coupling constants ($\lambda_{ij}^{0}$, where $\lambda_{11}^{0}$ and $\lambda_{22}^{0}$ are intraband and $\lambda_{12}^{0}$ and $\lambda_{21}^{0}$ are interband); label 1 relates to a band with “strong” SC condensate, and label 2 relates to one where the “weak” SC condensate develops, with band diffusivities of $D_{1}$ and $D_{2}$. In order to reduce the number of free parameters, we used the same coupling constants as in [26]: $\lambda_{11}=\lambda_{22}^{0}=1$, $\lambda_{11}^{0}\lambda_{22}^{0}-\lambda_{12}^{0}\lambda_{21}^{0}=0.5$. For $H_{c2}^{⊥}$, the resulting diffusivity ratio ($\frac{D_{1}}{D_{2}}\approx 6$) does not change for the three different $T_{c}$ criteria. This value is higher than the $\frac{D_{1}}{D_{2}}\approx 2.5$ obtained in [26] for the sample with 2% Co concentration, for which our $\frac{D_{1}}{D_{2}}\approx 6$ satisfies the general increasing tendency of the diffusivity ratio with doping observed in Figure 5b in [26].

However, for $H_{c2}^{\|}$, the two-band model can fit data by setting $D_{1}=D_{2}$, similarly to that obtained in [26]. In Figure 5d, we present the $H_{c2}\left(T\right)$ fit using two sets of full coupling constants estimated in the IMARE experiment (corresponding to different Coulomb repulsion strengths): set a: $\lambda_{11}^{0}=0.435$, $\lambda_{22}^{0}=0.326$, $\lambda_{12}^{0}=0.236$, and $\lambda_{21}^{0}=0.133$ (solid line); set b: $\lambda_{11}^{0}=0.412$, $\lambda_{22}^{0}=0.304$, $\lambda_{12}^{0}=0.219$, and $\lambda_{21}^{0}=0.108$ (dotted line) (see Section 4). In the latter case, the determined diffusivity ratios are $\frac{D_{1}}{D_{2}}\approx 3.52$ and 3.75, respectively.

Both single-band and two-band fits describe the experimental data quite well (see Figure 4 and Figure 5). Nevertheless, the determined $H_{c2}^{c}\left(0\right)$ value depends on the model (about 15%). The obtained estimates for the upper critical fields ($H_{c2}^{c}\left(0\right)$, $H_{c2}^{ab}\left(0\right)$) and slopes ($-\frac{dH_{c2}^{c}}{dT}{|}_{T_{c}}$, $-\frac{dH_{c2}^{ab}}{dT}{|}_{T_{c}})$ are given in Table 1. The resulting $-\frac{dH_{c2}^{c}}{dT}{|}_{T_{c}}\approx 2.5–2.7$ T/K are very close to the related values determined in [25,26] for 2.5% and 2% Co samples, respectively, whereas our $-\frac{dH_{c2}^{ab}}{dT}{|}_{T_{c}}\approx 6.3–7.0$ T/K values appear to be a slightly lower than that reported in [26] but higher that reported in in [25]. The resulting anisotropy of the upper critical field in the vicinity of $T_{c}$ is $\gamma_{H}\left(T\to T_{c}\right)\equiv \frac{H_{c2}^{ab}}{H_{c2}^{c}}\approx 2.5–2.8$ and resembles the $\gamma_{H}\left(T\to T_{c}\right)\approx 3$ value obtained in [26] and exceeds the $\gamma_{H}\left(T\to T_{c}\right)\approx 2.25$ estimated in [25] for an underdoped NaFe${}_{1-x}$Co${}_{x}$As crystal of similar composition.

For both field directions, since the temperature associated with a 50% R(T) drop almost coincides with that corresponding to max dR/dT, these two criteria of $T_{c}$ determination yield similar $H_{c2}\left(0\right)$ values, both lower than the $H_{c2}\left(0\right)$ estimated using the “$T_{c}^{\mathrm{onset}}$ criterion”. Generally, the temperature attributed to dR(T)/dT maximum indicates the transition of the main volume of the crystal to the SC state. Therefore, the $H_{c2}\left(0\right)$ values determined using the “max dR/dT” criterion seem to be physically correct.

The single-band WHH estimate provides the range of zero-temperature values between $\mu_{0}H_{c2}^{c}\left(0\right)\approx$ 30–36 T (depending on the chosen criterion) and $\mu_{0}H_{c2}^{ab}\left(0\right)\approx$ 54–60 T. The small increase in $H_{c2}^{ab,c}\left(0\right)$ estimated using the two-band fit as compared to that obtained by the single-band WHH model is caused by a “weak” SC condensate contribution to the upper critical field at low temperatures.

### 3.2. IMARE Spectroscopy

**Figure 6:**
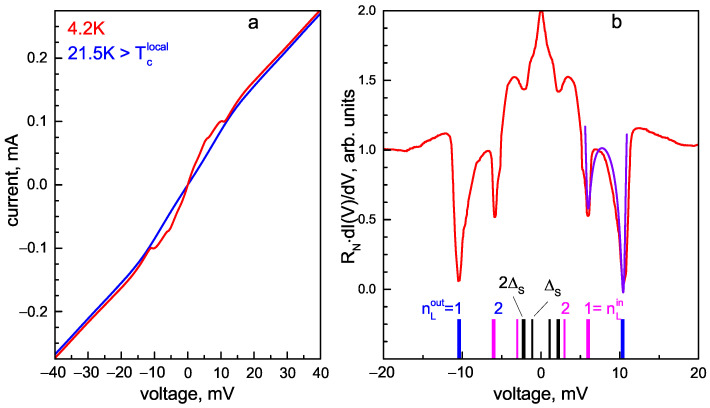
(**a**) Current–voltage characteristic of SnS junction in underdoped NaFe${}_{1-x}$Co${}_{x}$As at temperatures below and above $T_{c}$ (red and blue lines, respectively). (**b**) Corresponding dynamic conductance spectrum measured at T=4.2 K. The monotonic background is suppressed for clarity. Vertical bars show the positions of the Andreev features of $\Delta_{L}^{\mathrm{out}}\left(0\right)\approx 5.2$ meV (blue bars, $n_{L}^{\mathrm{out}}=1,\;2$ labels), $\Delta_{L}^{\mathrm{in}}\approx 3.1$ meV (magenta bars, $n_{L}^{\mathrm{in}}=1,\;2$ labels), and the small SC gap of $\Delta_{S}\left(0\right)\approx 1.1$ meV (black bars). The violet line fits the shape of the doublet with model presented in [34] for a case of extended *s*-wave symmetry of the large SC gap ($\Delta_{L}$).

Figure 6a shows the CVC of MCPBJ contact measured in the SC and the normal states at T=4.2 K and 21.5 K, respectively. The local critical temperature of this junction corresponding to the contact area transition to the normal state is $T_{c}\approx 19.8$ K (see below). Below $T_{c}$, the supercurrent branch at eV=0 is absent in the CVC; instead, a foot area with enhanced dynamic conductance appears in the corresponding dI(V)/dV spectrum shown in Figure 6b. At 4.2 K (red curve in Figure 6a), an excess current presents in the CVC as compared to that above $T_{c}$ (blue line), tending toward a constant value at high bias voltages. The observed CVC features at $T\ll T_{c}$ are typical for a “long” SnS junction with incoherent transport and high transparency of the barrier, in accordance with all theoretical models describing the multiple Andreev reflection effect [28,29,30,31,32,33].

In the normal state, all the I(V) features caused by IMARE vanish, but the CVC remains slightly nonlinear, showing the unconventional nature of NaFe${}_{1-x}$Co${}_{x}$As. The resembling I(V) nonlinearity is reproducible and cannot be caused by a junction overheating during the current flow or any geometrical resonance, since a random contact dimension is obtained by the MCPBJ technique (for details, see Figure 1 in [45]). On the contrary, the nonlinear I(V) normal-state behavior may originate from the features of the electron density of N(E) states in the vicinity of the Fermi level [46] caused by either specific band structure topology or resonant electron–boson interaction, which requires further studies.

The corresponding dI(V)/dV spectrum measured at $T\ll T_{c}$ (Figure 6b) shows a series of dips. The pronounced minima located at ∣eV∣≈10.4 and 6.2 meV do not satisfy the subharmonic positions as n=1,2 or n=2,3 numbers and therefore cannot be attributed to one and the same SGS caused by a single SC gap. At higher bias voltages, the dI(V)/dV spectrum has no features caused by the SC state. Hence, we consider this doublet as the fundamental (n=1) Andreev feature, the position of which directly determines two SC-order parameters: $2\Delta_{L}^{\mathrm{out}}\approx 10.4$ meV and $2\Delta_{L}^{\mathrm{in}}\approx 6.2$ meV (bold blue and magenta bars, respectively; $n_{L}^{in,out}$ labels in Figure 6b). A minor feature at ∣eV∣≈5.2 meV could be interpreted as a second subharmonic ($n_{L}^{\mathrm{out}}=2$) of $\Delta_{L}^{\mathrm{out}}$. The position of the $n_{L}^{}=2$ Andreev feature caused by the $\Delta_{L}^{\mathrm{in}}$ order parameter expected at ∣eV∣≈3.1 meV is not resolved, since it merges with the fundamental harmonic ($n_{S}=1$) of the small SC gap ($2\Delta_{S}\approx 2.2$ meV) and therefore possibly smears it. At lower bias voltages, the second $\Delta_{S}$ subharmonic is also visible (thin black bars, $\Delta_{S}$ labels in Figure 6b).

The typical arch-like shape of the doublet could be fitted within the approach proposed in [34] for a case of SC gap with extended *s*-wave symmetry of cos(4θ) type without nodes (compare the experimental dI(V)/dV and the blue line fit in Figure 6b). Therefore, the observed doublet could be interpreted as a wide Andreev feature of a single SC-order parameter developing below $T_{c}$ on one and the same Fermi surface sheet with a moderate anisotropy in the *k* space. In this very probable case, the directly determined SC energy parameters ($\Delta_{L}^{\mathrm{in}}$ and $\Delta_{L}^{\mathrm{out}}$) could be considered as the gap edges; the minimum and maximum Cooper pair coupling energies in this band depend on the momentum direction in the $k_{x}k_{y}$ plane. The less probable scenario is that the determined $2\Delta_{L}^{\mathrm{out}}$ and $2\Delta_{L}^{\mathrm{in}}$ could represent two distinct SC-order parameters characterizing the properties of two SC condensates developed in different bands (see Section 4).

**Figure 7:**
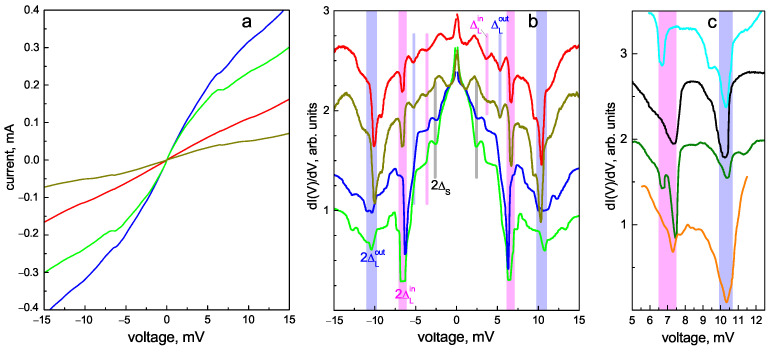
(**a**) Current–voltage characteristics of SnS junctions with different normal resistance ($R_{N}$) values at T=4.2 K obtained in various underdoped NaFe${}_{1-x}$Co${}_{x}$As single crystals from the same batch. (**b**) Corresponding dynamic conductance spectra measured at T=4.2 K. Vertical blue and magenta bars show the positions of the Andreev dips of $2\Delta_{L}^{\mathrm{out}}\left(0\right)\approx 10.4$ meV and $2\Delta_{L}^{\mathrm{in}}\approx 6.6$ meV, respectively. Gray lines point to the fundamental Andreev dip of the small SC gap ($2\Delta_{S}\left(0\right)\approx 2.4$ meV). (**c**) Fragments of dI(V)/dV spectra for various contacts measured at T=4.2 K, reproducibly showing a doublet Andreev feature of the large SC gap.

The complex structure observed in the dI(V)/dV characteristics of SnS junctions with random geometry is reproducible. Figure 7a,b show a set of CVCs and the corresponding dynamic conductance spectra of SnS junctions with normal resistances of $R_{N}\approx$ 50–175 Ohm formed in the single crystals from the same batch. The $R_{N}$ value depends on the contact dimensions and transparency of the barrier, and can generally change from one contact point to another. Despite such a wide $R_{N}$ range, the positions of all IMARE features in the corresponding dI(V)/dV spectra remain almost constant. In Figure 7b, the positions of the doublet (blue and magenta bars, $2\Delta_{L}$ labels) directly determine the SC-order parameters ($2\Delta_{L}^{\mathrm{out}}\left(0\right)\approx 10.4$ meV and $2\Delta_{L}^{\mathrm{in}}\left(0\right)\approx 6.6$ meV). In the two upper spectra in Figure 7b, the second (n=2) Andreev subharmonic also forms a well-resolved doublet (thin blue and magenta bars, $\Delta_{L}$ labels). According to the formula of SGS, it is located at a position two times smaller than that of the fundamental one. The Andreev feature of the small SC gap is present in the two bottom curves as a dip located at ∣eV∣≈2.4 meV and corresponds to the $2\Delta_{S}\left(0\right)\approx 2.4$ meV magnitude. This $2\Delta_{S}$ feature is possibly undistinguished in the red and dark yellow upper spectra in Figure 7b due to its merging with the SGS of $\Delta_{L}^{\mathrm{in}}$. A representative set of the dI(V)/dV arch-like doublets (of the fundamental n=1 feature) obtained in different underdoped NaFe${}_{1-x}$Co${}_{x}$As single crystals from the same batch is shown in Figure 7c.

**Figure 8:**
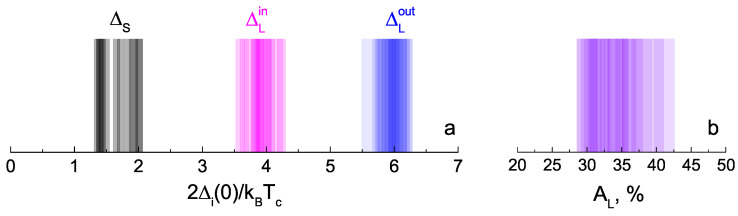
(**a**) Color histogram of the characteristic ratios ($2\Delta_{i}\left(0\right)/k_{B}T_{c}$) of the determined SC-order parameters ($\Delta_{L}^{\mathrm{out}}$, $\Delta_{L}^{\mathrm{in}}$, and $\Delta_{S}$) at $T\ll T_{c}$. The data were obtained with underdoped NaFe${}_{1-x}$Co${}_{x}$As single crystals from the same batch, and the range of local critical temperatures of the SnS junctions was 19–22 K. Each value is shown by a semitransparent bar (blue, magenta, or black), whereas the position (along the horizontal axis) of the most intense color corresponds frequency of experimental attainment. (**b**) Color histogram for the possible $\Delta_{L}$ anisotropy value taken as $A_{L}\equiv 100\%\cdot \left[1-\Delta_{L}^{\mathrm{in}}/\Delta_{L}^{\mathrm{out}}\right]$.

The large IMARE data obtained with underdoped NaFe${}_{1-x}$Co${}_{x}$As are shown in Figure 8. The presented data involve the energy parameters of SnS junctions with local critical temperatures $T_{c}\approx 19–22$ K (the temperature of the contact area transition to the normal state). In order to account for the $\Delta_{i}$ variation within the obtained $T_{c}$ range, the values of characteristic ratios $2\Delta_{i}\left(0\right)/k_{B}T_{c}$ are compared and summarized in color histogram (Figure 8a). The data are presented as semitransparent bars positioned along the horizontal axis corresponding to the characteristic ratios of all SC-order parameters obtained by us (the vertical axis does not matter), whereas the area of the most intensive color points corresponds to the most frequently observed experimental value. For the two largest SC-order parameters, the characteristic ratios are $2\Delta_{L}^{\mathrm{out}}\left(0\right)\approx 6.0\pm 0.3$ and $2\Delta_{L}^{\mathrm{in}}\left(0\right)/k_{B}T_{c}\approx 3.9\pm 0.5$. Supposing $\Delta_{L}^{\mathrm{in},\mathrm{out}}$ as the edges of one and the same anisotropic SC-order parameter $\Delta_{L}$, its possible anisotropy in the momentum space can be estimated as $A_{L}\approx 28–43\%$ (Figure 8b). For the small SC gap, the characteristic ratio ($2\Delta_{S}\left(0\right)/k_{B}T_{c}\approx 1.4–2.0$) appears below the weak coupling BCS limit 3.5, which is typical for a “weak” SC condensate in a multiple-gap superconductor.

**Figure 9:**
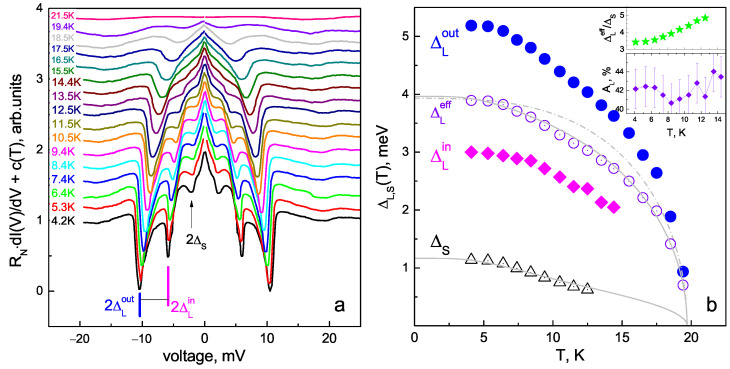
(**a**) Evolution of the dI(V)/dV spectrum shown in Figure 6 with temperature. The curves are manually shifted along the vertical axis for clarity by c(T). (**b**) Temperature dependences of the SC-order parameters ($\Delta_{L}^{\mathrm{out}}\left(T\right)$, $\Delta_{L}^{\mathrm{in}}\left(T\right)$, and $\Delta_{S}\left(T\right)$) directly determined using panel data (**a**) (solid circles, rhombs, and open triangles, respectively). The effective large SC gap determined as $\Delta_{L}^{\mathrm{eff}}\equiv \sqrt{\Delta_{L}^{\mathrm{out}}\cdot \Delta_{L}^{\mathrm{in}}}$ is shown by open circles. Single-band BCS-like behavior (dash–dot line) and two-band fits using Moskalenko and Suhl equations with renormalized BCS integrals (gray solid line) are shown for comparison. **The upper inset** presents the ratio between $\Delta_{L}^{\mathrm{eff}}$ and $\Delta_{S}$ vs. temperature, and the **lower inset** shows the temperature dependence of the possible anisotropy of $\Delta_{L}$.

Consider the temperature evolution of the SC gap structure in the dI(V)/dV spectrum of the SnS junction (as presented in Figure 6) shown in Figure 9a. The dI(V)/dV curves shown in Figure 9a are manually offset vertically for clarity, whereas the normal resistance ($R_{N}$) of the junction remains almost constant, with the temperature pointing to ballistic transport through this junction (compare the red and blue CVCs parallel at eV≫2Δ(0) in Figure 6a). With increased temperature, the zero-bias conductance peak shrinks, whereas all the Andreev features become less intense and shift toward zero bias, driven by the reduction in the number of Cooper pairs. At $T\approx 21.5\;K>T_{c}$ (upper pink curve in Figure 9a), all the IMARE-caused features vanish in the dI(V)/dV spectrum, indicating the transition of the contact area to the normal state and the absence of Cooper pairs.

Temperature dependences ($\Delta_{L}^{\mathrm{in},\mathrm{out}}\left(T\right)$) directly determined using the evolution of the dI(V)/dV doublet position are shown in Figure 9b by solid magenta rhombs and blue circles, respectively. The lower inset presents the temperature dependence of the possible large gap anisotropy: $A_{L}\approx 42\%\approx const$ hardly evolves with temperature, which results in quite similar temperature behavior of $\Delta_{L}^{\mathrm{in}}$ and $\Delta_{L}^{\mathrm{out}}$. In order to designate the effective value of the large SC gap, we take its zero-temperature value as $\Delta_{L}^{\mathrm{eff}}\left(0\right)\equiv \sqrt{\Delta_{L}^{\mathrm{in}}\left(0\right)\Delta_{L}^{\mathrm{out}}\left(0\right)}$ and associate it with the $\delta_{L}^{\mathrm{out}}\left(T\right)$ temperature trend (δ(T)≡Δ(T)/Δ(0)); the resulting $\Delta_{L}^{\mathrm{eff}}\left(T\right)$ dependence is shown in Figure 9b by open violet circles. Starting from T≈ 5–6 K, the small SC gap decreases a slightly more rapidly compared to the large one. The $\Delta_{L}^{\mathrm{eff}}\left(T\right)/\Delta_{S}\left(T\right)$ ratio increases with temperature, as shown by the green stars in the upper inset of Figure 9b. Due to differing temperature behavior, we attribute the corresponding low-bias dI(V)/dV features as Andreev subharmonics of a distinct $\Delta_{S}$-order parameter.

The dependence of $\Delta_{L}^{\mathrm{eff}}$ on temperature roughly resembles a standard single-band BCS-like function (dash–dot line in Figure 9b) but slightly bends down at T≈ 5–18 K. The small SC gap shows more significant curving, which is typical for a multiple-gap SC with moderate interband coupling (comparable to intraband coupling). As a rough estimate, the $\Delta_{L}^{\mathrm{eff}}\left(T\right)$ and $\Delta_{S}\left(T\right)$ temperature dependences were fitted using a two-band model based on Moskalenko and Suhl et al.’s system of equations [47,48,49] with renormalized temperatures in two BCS integrals [50]. We used the experimental values of $T_{c}$ and $\Delta_{i}\left(0\right)$ and the two free parameters: $\alpha =\lambda_{12}/\lambda_{21}$, $\beta =\sqrt{\lambda_{11}\lambda_{22}}/\sqrt{\lambda_{12}\lambda_{21}}$; other parameters were calculated using the equations mentioned above. The resulting fits are in agreement with the experimental data (solid gray lines in Figure 9b).

Using a two-band fit, the estimated quadruple of renormalized or “weak” coupling constants is $\lambda_{11}=0.292$, $\lambda_{22}=0.184$, $\lambda_{12}=0.098$, $\lambda_{21}=0.022$. Due to abundance of free parameters, extraction of full coupling constants ($\lambda_{ij}^{0}$) becomes ambiguous.

## 4. Discussion

Since the Andreev features of $\Delta_{S}$ are located on the notably sloped dI(V)/dV background (at the foot area caused by $\Delta_{L}$ condensate), determination of its exact positions is complicated, resulting in increased diversity of the corresponding characteristic ratio as compared to that of the large SC gaps. Another reason for this could be the possible anisotropy of the small SC gap. No clear doublets of $\Delta_{S}$ are reproducibly observed in the dI(V)/dV spectra, which could be a sign of either the *s*-wave symmetry type of the small SC gap, its strong anisotropy ($A_{S}>50\%$), or even nodal distribution ($A_{S}=100\%$) in the momentum space. Since the ZBC value is determined by the structure of both SC-order parameters, it is impossible to make a judgment about the presence of nodes for a small gap.

**Figure 10:**
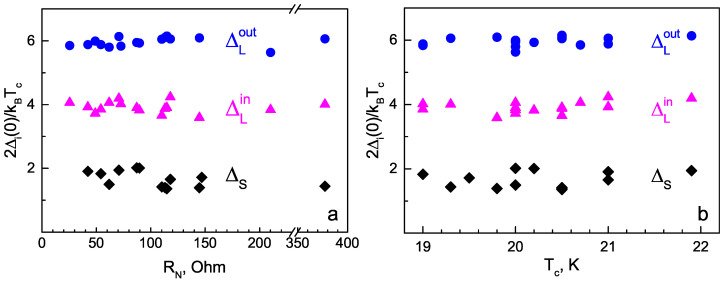
The dependences of the SC-order parameter characteristic ratios on the normal resistance ($R_{N}$) of contacts under study (**a**) and on the local critical temperature ($T_{c}$) of the junction (**b**).

All the determined $2\Delta_{i}\left(0\right)/k_{B}T_{c}$ values are reproducible and do not depend on the normal resistance of the corresponding SnS junction (which is random), as shown in Figure 10a. Therefore, the energy parameters directly determined in our IMARE experiment do not depend on the contact geometry, and the proportion of ballistic-to-diffusive transport reflects the bulk properties of the SC subsystem of NaFe${}_{1-x}$Co${}_{x}$As and cannot be attributed to dimensional effects or artefacts. Accordingly, the characteristic ratios for the three SC-order parameters remain almost independent at the local critical temperature within the studied $T_{c}\approx$ 19–22 K range (Figure 10b), which indicates a scaling between $\Delta_{L}^{\mathrm{out}}$, $\Delta_{L}^{\mathrm{in}}$, and $\Delta_{S}$ with local $T_{c}$.

Noteworthily, in the IMARE experiment, only the energy parameters ($\Delta_{i}$) of the SC state and their temperature dependences were directly determined, rather than the symmetry type or sign of the SC gap. As mentioned above, the arch-like doublets reproducibly observed in the dI(V)/dV spectra could be caused by a single $\Delta_{L}$ SC gap with *k*-space anisotropy. Less probable is the possibility of two isotropic SC gaps ($\Delta_{L}^{\mathrm{in}}$ and $\Delta_{L}^{\mathrm{out}}$) developing in different bands. In order to directly distinguish between the abovementioned possibilities, additional theoretical and experimental studies are necessary. Therefore, the possible anisotropy of the large SC gap remains ambiguous.

Nonetheless, several issues can be highlighted that favor the anisotropy scenario. First, as mentioned above, the SC gap anisotropy is generally supposed for NaFe${}_{1-x}$Co${}_{x}$As, as well as for other families of iron-based superconductors in the framework of both the $s^{\pm }$ and $s^{++}$ approaches [17,18,20]. Secondly, the shape of the doublet representing two dips connected by an arch resembles that numerically calculated for an SC-order parameter with extended *s*-wave symmetry of the cos(4θ) type without nodes (violet line fit in Figure 6c; see also the magenta theoretical curve in Figure A1b). Moreover, the relative width of the doublet remains almost constant with temperature (see the inset of Figure 9b), providing $\Delta_{L}^{\mathrm{in}}\left(T\right)/\Delta_{L}^{\mathrm{out}}\left(T\right)\approx const$.

This behavior resembles the temperature evolution of similar dynamic conductance doublets reproducibly observed by us earlier in sister LiFeAs compound [51], as well as BaFe${}_{2-x}$Ni${}_{x}$As pnictides [52]. Generally, in a multiple-gap SC, the $\Delta_{1}\left(T\right)/\Delta_{2}\left(T\right)\approx const$ behavior takes place in the only case of $\det(\lambda_{ij})=0$, yielding β=1. Since the realization of this exact case in NaFe${}_{1-x}$Co${}_{x}$As and the abovementioned LiFeAs and BaFe${}_{2-x}$Ni${}_{x}$As seems less probable, one could consider the observed $A_{L}\approx const$ dependence as favoring our suggestion about the realization of *k*-space anisotropy of the large SC gap. Additionally, the estimated $A_{L}\approx$ 28–43% is close to the large SC gap anisotropy (≈30%) determined by ARPES [12].

Since electron–phonon coupling seems rather weak in Fe-based superconductors [53], we did not use the phonon renormalization term $\left(1+\lambda_{ij}\right)$, determining $\lambda_{ij}^{0}=\lambda_{ij}+\mu_{ij}^{*}$, where $\mu_{ij}^{*}$ are Coulomb pseudopotentials. The cutoff energy was taken as $\omega_{c}=40$ meV. As a result, one could obtain two sets of full coupling constants: set (a): $\lambda_{11}^{0}=0.412$, $\lambda_{22}^{0}=0.304$, $\lambda_{12}^{0}=0.219$, and $\lambda_{21}^{0}=0.108$ for a weak Coulomb repulsion with free parameter $\mu_{eff}^{*}=0.112$; set (b): $\lambda_{11}^{0}=0.435$, $\lambda_{22}^{0}=0.326$, $\lambda_{12}^{0}=0.236$, and $\lambda_{21}^{0}=0.133$ (the used coupling constant sets are labeled as $\lambda_{\mathrm{IMARE}}^{0}$ in Figure 5d) for a moderate Coulomb repulsion with a reasonable $\mu_{eff}^{*}=0.134$ (this value is similar to that calculated in [54] for NaFeAs). Free choice of $\mu^{*}>0.2$ seems hardly eligible unless there is a specific reason. For the two obtained quadruples of $\lambda_{ij}^{0}$, the estimated ratio between the normal-state band density of state $N_{i}$ near the Fermi level are $\alpha \equiv N_{2}/N_{1}\approx 2$ and 1.8, and the intraband-to-interband coupling strengths are β≈2.3 and 2.1 for sets (a,b), respectively.

The two obtained sets of full coupling constants were used to fit the upper critical field temperature dependence with the two-band Gurevich model [44]. As experimental data, the $\mu_{0}H_{c2}^{⊥}\left(T\right)$ dependence obtained with the maxdR/dT criterion was used as follows. First, the corresponding $T_{c}$ coincides with the local critical temperature of the studied SnS junction shown in Figure 9. Secondly, since our IMARE experiment with planar junctions generally provides information about the SC gap anisotropy in the $k_{x}k_{y}$-plane, the obtained SC properties should be compared with the upper critical field ($\mu_{0}H_{c2}^{⊥}\left(T\right)$), which is determined by the in-plane coherence length ($\xi_{ab}$). Both $\lambda_{\mathrm{IMARE}}^{0}$ sets can fit the linear $\mu_{0}H_{c2}^{⊥}\left(T\right)$ fading in the vicinity of $T_{c}$ and yield a zero temperature value ($\mu_{0}H_{c2}^{⊥}\left(0\right)\approx 42$ T). The strength of Coulomb repulsion does not seriously affect the resulting fit.

## 5. Conclusions

Underdoped NaFe${}_{0.979}$Co${}_{0.021}$As SC pnictide single crystals with a critical temperature of $T_{c}\approx 20.5$ K were grown using a “self-flux” technique. Using magnetotransport probes, the temperature dependences of the upper critical field ($H_{c2}\left(T\right)$) were measured within the range of H≤16 T in two field orientations, and the slopes ($-\frac{dH_{c2}^{ab}}{dT}{|}_{T_{c}}\approx$ 6.34–6.97 and $-\frac{dH_{c2}^{c}}{dT}{|}_{T_{c}}\approx$ 2.45–2.74 (depending on the $T_{c}$ criterion)) were determined. The experimental $H_{c2}\left(T\right)$ dependences can be fitted with both the WHH and Gurevich models, with estimated $H_{c2}^{ab}\left(0\right)$ and $H_{c2}^{c}\left(0\right)$ up to 77 and 59 T, respectively (using the two-band approach).

Using IMARE spectroscopy of SnS junctions made using the MCPBJ technique, the magnitudes and temperature dependences of the SC-order parameters ($\Delta_{L,S}\left(T\right)$) were determined locally and directly. A possible $A_{L}\approx$ (28–43)% anisotropy of the large SC gap was shown in the *k* space, which remained almost constant with temperature up to $T_{c}$.

The obtained characteristic ratio for the large SC gap is $2\Delta_{L}\left(0\right)/k_{B}T_{c}\approx$ 3.9–6.0 (the range corresponds to the possible $\Delta_{L}$ anisotropy), whereas that for the small SC gap is $2\Delta_{S}\left(0\right)/k_{B}T_{c}\approx$ 1.4–2.0 < 3.5, which is typical for a “weak” SC condensate in a multiple-band superconductor. Using two-band fit of the experimental $\Delta_{L,S}\left(T\right)$ curves, we estimated a quadruple of $\lambda_{ij}^{0}$ coupling constants and showed that it could be used in order to quantitatively fit the experimental $H_{c2}\left(T\right)$ with a two-band model, yielding a band diffusivity ratio of $D_{1}/D_{2}\approx 3.5–3.8$.

## Figures and Tables

**Table 1:** Summary of parameters for the upper critical field ($H_{c2})$ of the investigated NaFe${}_{0.979}$Co${}_{0.021}$As: $H_{c2}\left(T\right)$ slopes at $T\to T_{c}$, H‖ab, and H‖c directions; magnetic anisotropy $\gamma_{H}\left(T\to T_{c}\right)\equiv H_{c2}^{ab}/H_{c2}^{c}$; estimated zero-temperature values of $H_{c2}\left(0\right)$ (where I and II correspond to the one-band and two-band model, respectively); and coherence lengths $\xi_{ab,c}$.

Criterion	$-\frac{{\mathrm{dH}}_{c2}^{\mathrm{ab}}}{\mathrm{dT}}{|}_{T_{c}}$	$-\frac{{\mathrm{dH}}_{c2}^{c}}{\mathrm{dT}}{|}_{T_{c}}$	$\gamma_{H}\left(T_{c}\right)$	$H_{c2}^{I,c}\left(0\right)$	$H_{c2}^{\mathrm{II},c}\left(0\right)$	$H_{c2}^{I,\mathrm{ab}}\left(0\right)$	$H_{c2}^{\mathrm{II},\mathrm{ab}}\left(0\right)$	$\xi_{\mathrm{ab}}\left(0\right)$	$\xi_{c}\left(0\right)$
	T/K	T/K		T	T	T	T	nm	nm
50%	6.85	2.45	2.8	30	49	58	61	2.59	2.08
max dR/dT	6.34	2.52	2.5	32	52	54	60	2.51	2.18
$T_{c}^{onset}$	6.97	2.74	2.54	36	59	60	77	2.36	1.81

## Data Availability

The experimental data are available on demand.

## Abbreviations

The following abbreviations are used in this manuscript:

SC	Superconducting
XRD	X-ray diffraction
BCS	Bardeen–Cooper–Schrieffer
WHH	Werthammer–Helfand–Hohenberg
AFM	Antiferromagnetism
ARPES	Angle-resolved photoemission spectroscopy
CVC	Current–voltage characteristic
IMARE	Incoherent multiple Andreev reflection effect
MCPBJ	Mechanically controlled planar break junction
SGS	Subharmonic gap structure
SnS	Superconductor–normal metal–superconductor
ZBC	Zero-bias conductance

## Appendix A. IMARE Spectroscopy Details

Figure A1a shows the theoretical CVC and dI(V)/dV of an SnS junction (based on a single-gap BCS superconductor) predicted by [28]. The data are calculated for a semiballistic regime ($l^{\mathrm{el}}/d<2$).

During IMARE, incoherent transport through the SnS junction (“long” contact regime) causes no supercurrent branch at eV=0 in the CVC, contrary to the case of a “short” junction with d<ξ and therefore with phase coherence between the SC banks, as described in [32,33]. Additionally, in the IMARE regime, an excess current ($I_{\mathrm{exc}}$) appears within the whole bias voltage range, as compared to CVC in the normal state above $T_{c}$ [28,29,30,31]. At high bias voltages, $I_{\mathrm{exc}}\left(V\right)$ tends toward a constant value. In the case of a highly transparent metallic layer (transparency >80%, barrier strength Z<0.35), dynamic conductance drastically rises at eV→0, forming a so-called “foot” area and an enhanced ZBC peak at eV→0 in the dI(V)/dV spectrum [28,30,31].

A series of SGS features at positions $eV_{n}\left(T\right)=2\Delta \left(T\right)/n$ (n=1,2,…) also appears in the I(V) and dI(V)/dV curves of the SnS junction below $T_{c}$ [28,29,32,33]. For a highly transparent junction (typically observed in Na(Fe,Co)As (see Figure 6 and Figure 7)), SGS represents a series of dI(V)/dV dips (the dashed line in Figure A1a). The number ($n^{*}$) of observable SGS dips depends mainly on the ballistic $l^{\mathrm{el}}/d$ ratio [30,31]. Even minor occurrence of normal reflections (resulting in *Z* increase) and inelastic scattering reduce $n^{*}$. As a result, for the studied SnS junctions, one to two harmonics are visible in the dI(V)/dV: a fundamental one at eV=2Δ and a second one at eV=Δ.

With a temperature increase, all the IMARE features ($I_{\mathrm{exc}}$, ZBC, and SGS) become less intensive and fully vanish at $T_{c}$. The position of the SGS shifts toward zero bias in accordance with the Δ(T) temperature dependence (see solid lines in Figure A1a).

The shape of the SGS features in the dI(V)/dV curve strongly depends on the SC gap symmetry. Figure A1b shows typical Andreev dips calculated numerically in the framework proposed in [34]. In the case of an isotropic SC gap with *s*-wave symmetry type, IMARE causes sharp and pronounced Andreev dips in the dI(V)/dV spectrum (black line). For SC-order parameters with any nodal angle distribution (Δ(θ), where θ is an angle in the momentum space), including the *d*-wave symmetry type, a strong suppression of the SGS dip intensity is expected (note, the amplitude of the blue line in Figure A1 is manually zoomed by a factor of 10 along the vertical axis for clarity). In the case of an anisotropic SC-order parameter with extended *s*-wave symmetry, each SGS subharmonic would represent a doublet. As an example, the angle distribution (Δ(θ)) of an SC gap with 30% anisotropy in the $k_{x}k_{y}$ plane (in the inset) and the corresponding doublet are shown in Figure A1 (magenta line).

In Figure A1c, a scheme of MCPBJ contact in the layered single crystal is presented. For such compounds, steps and terraces naturally appear on the cryogenic clefts (shown by gray areas on the top of Figure A1c). By gently tuning the sample holder curvature, the cryogenic clefts slide onto each other along the ab direction, touching through various terraces. As a result, the area (determining the normal resistance ($R_{N}$)) of the junction rather than the distance between the SC clefts (and therefore the barrier strength (*Z*)) is the adjustable parameter in our experimental setup. According to our estimates, the typical size of the MCPBJ contact is about 10–50 nm along the ab plane [51,52].

Generally, the crack is located in the bulk of the sample and far from current and potential leads. The MCPBJ contact is not fully opened, preventing the penetration of impurities into the crack and degradation of the superconducting properties on their surface, in addition to minimizing overheating of the junction during the current flow. For more details, see [36].
